# Bone marrow mesenchymal cells: polymorphism associated with transformation of rough endoplasmic reticulum

**DOI:** 10.1097/BS9.0000000000000062

**Published:** 2020-11-17

**Authors:** Yong-Xin Ru, Shu-Xu Dong, Chun-Hui Xu, Shi-Xuan Zhao, Hua-Mei Zhang, Hao-Yue Liang, Min Fen, Feng-Kui Zhang, Ying-Dai Gao, Shu-Lin Qi, Hong-Cai Shang

**Affiliations:** a State Key Laboratory of Experimental Hematology, National Clinical Research Center for Blood Diseases, Institute of Hematology and Blood Diseases Hospital, Chinese Academy of Medical Sciences and Peking Union Medical College, Tianjin, China; b Key Laboratory of Chinese Internal Medicine of Ministry of Education, Beijing Dongzhimen Hospital, Beijing University of Chinese Medicine, Peking, China

**Keywords:** Bone-marrow mesenchymal cell, Morphology, Plasmalemma, Rough endoplasmic reticulum, Transformation

## Abstract

To understand the behavior and function of bone-marrow mesenchymal cells (BMMCs), we overviewed the morphological presentation of BMMCs in bone-marrow granules (b-BMMCs), isolated BMMCs (i-BMMCs), and BMMCs (c-BMMCs) cultured in H4434 methylcellulose semisolid and MEM media. All samples were derived from bone-marrow aspirates of 30 patients with hematocytopenia. Light microscopy exhibited b-BMMCs and i-BMMCs characterized by abundant cytoplasm and irregular shape in bone-marrow smears, as well as c-BMMCs in culture conditions. Scanning electron microscopy demonstrated cultured c-BMMCs with a sheet-like feature enveloping hematopoietic cells. Transmission electron microscopy revealed b-BMMCs constructing a honeycomb-like structure by thin bifurcate processes among hematopoietic cells. Furthermore, i-BMMCs had bifurcate parapodiums on the surface and prominent rough endoplasmic reticulum (rER) connected with the plasmalemma of the parapodiums. The detailed images suggested that rER may serve as a membrane resource for plasmalemmal expansion in BMMCs in bone marrow.

## INTRODUCTION

Bone-marrow mesenchymal cells (BMMCs) construct hematopoietic microenvironments to regulate hematopoietic stem/progenitor cells (HSPCs) in bone marrow. The morphologic relationship between BMMCs and HSPCs has been analyzed from the 1960s to 1990s,^1–4^ and the biological effects of BMMCs on HSPCs have been the object of intense study in recent decades.^5–7^ BMMCs participate in blood production through intimate cell-cell contacts and the release of hematopoietic factors.^8,9^ In a previous study, we described BMMCs characterized by fibroblastic and macrophage features constructing a hierarchical meshwork by divaricate processes in bone marrow.^10^ To understand the behavior and function of BMMCs, we examined BMMCs in bone-marrow granules and in culture by light microscopy, scanning and transmission electron microscopy (SEM and TEM). In the bone-marrow granules, which are regarded as having a good replication of the *in vivo* state of bone marrow, BMMCs within areas of organized bone-marrow tissue were designated as b-BMMCs, while solitary BMMCs also from bone-marrow granules but not closely associated with any other cells as in a tissue, were designated as i-BMMCs (“isolated” BMMCs). The i-BMMCs appear to have become detached as solitary cells during the aspiration process. BMMCs in culture were designated as c-BMMCs.

## RESULTS

### Morphology of b-BMMCs on light microscopy

**Figure 1:**
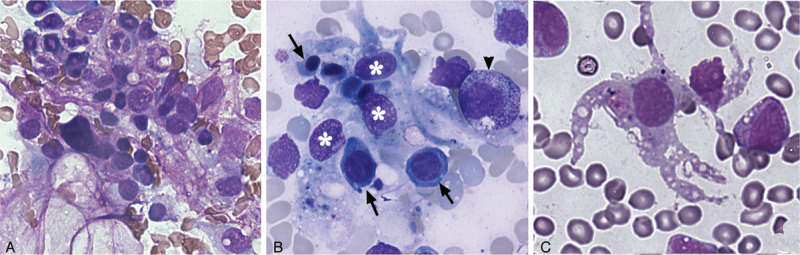
Characteristics of b-BMMCs in bone-marrow smears. (A) b-BMMCs are indiscernible in a large bone-marrow granule partly because of the overlapping of hematopoietic cells; (B) three b-BMMCs (stars) contacting erythrocytes (arrows) and granulocytes (arrowhead) are distinguishable in a small bone-marrow granule; (C) an isolated b-BMMC shows coarse cytoplasmic processes with vacuoles and granules.

Bone-marrow granules varied in size and contained many hematopoietic cells (erythrocytes and granulocytes) in bone-marrow smears. Most b-BMMCs were difficult to distinguish from hematopoietic cells and macrophages on account of cellular overlapping in large bone-marrow granules, but some b-BMMCs clearly exhibited a round nucleus and irregular shape with cytoplasmic processes around hematopoietic cells in small bone-marrow granules **(**Fig. 1**A, B**). Sometimes, i-BMMCs were found among hematopoietic cells in bone-marrow smears, characterized by a round nucleus, irregular processes with granular cytoplasm and vacuoles (Fig. 1**C**).

**Figure 2:**
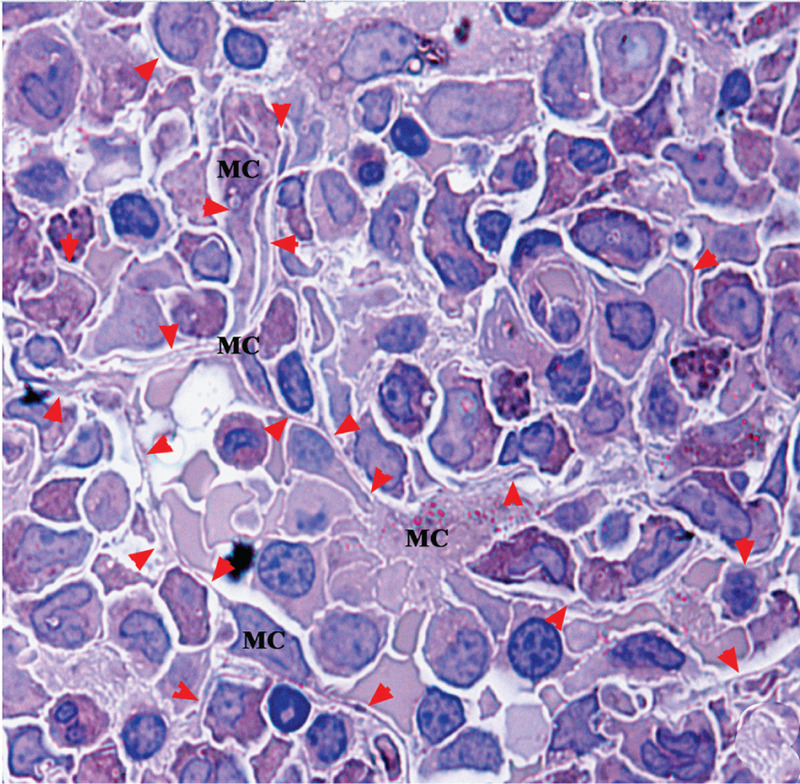
A bone-marrow granule in semithin sections stained with Wright-Giemsa. There are many b-BMMCs (MC) sending off thin bifurcate processes (arrowheads) among hematopoietic cells.

In semithin sections of Epon 812 blocks, b-BMMCs showed a main body with a nucleus and scanty cytoplasm, and long thin processes around hematopoietic cells. Proximate processes were usually thick and divaricated into thinner ones between hematopoietic cells. Additionally, there was a circular crevice between hematopoietic cells and processes. The circular crevices and processes constructed a single compartment for each hematopoietic cell (Fig. 2).

### Morphology of c-BMMCs on light microscopy

**Figure 3:**
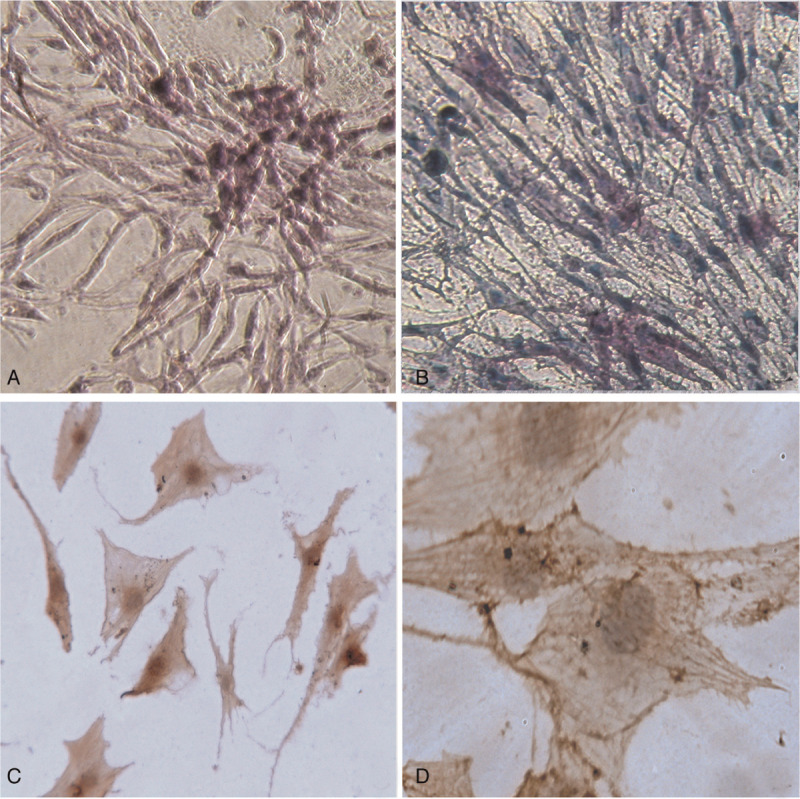
Characteristics of c-BMMCs in culture. (A) Wright staining shows a cluster of c-BMMCs attached to hematopoietic cells on the dish bottoms in H4434 methylcellulose semisolid medium; (B) some flat c-BMMCs show AKP activity in H4434 methylcellulose semisolid medium; (C) and (D) c-BMMCs in MEM media are flat and polygonal, and positive for CD44 and α-SMA respectively.

**Figure 4:**
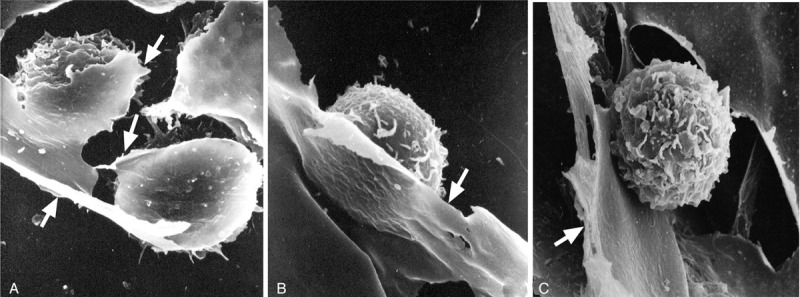
Scanning electron micrographs of c-BMMCs co-cultured with hematopoietic cells. (A) c-BMMCs (arrows) show a sheet-like feature enveloping 3 hematopoietic cells, ×2.5K; (B) sheet-like process (arrow) supports a hematopoietic cell, ×3K; (C) a hematopoietic cell is isolated by c-BMMCs (arrow), ×3K.

Clusters of c-BMMCs were scattered over the bottoms of dishes in H4434 methylcellulose semisolid medium. Most were spindle shaped, while some, adherent to hematopoietic cells, were flat and showed AKP activity on inverted microscopy (Fig. 3**A, B**). On the other hand, c-BMMCs in MEM media were flat and polygon-like, and positive for CD44 and α-SMA (Fig. 3**C, D**). By SEM, these c-BMMCs looked like a huge thin blanket, enveloping and supporting blood cells (Fig. 4).

### Ultrastructure of b-BMMCs on TEM

**Figure 5:**
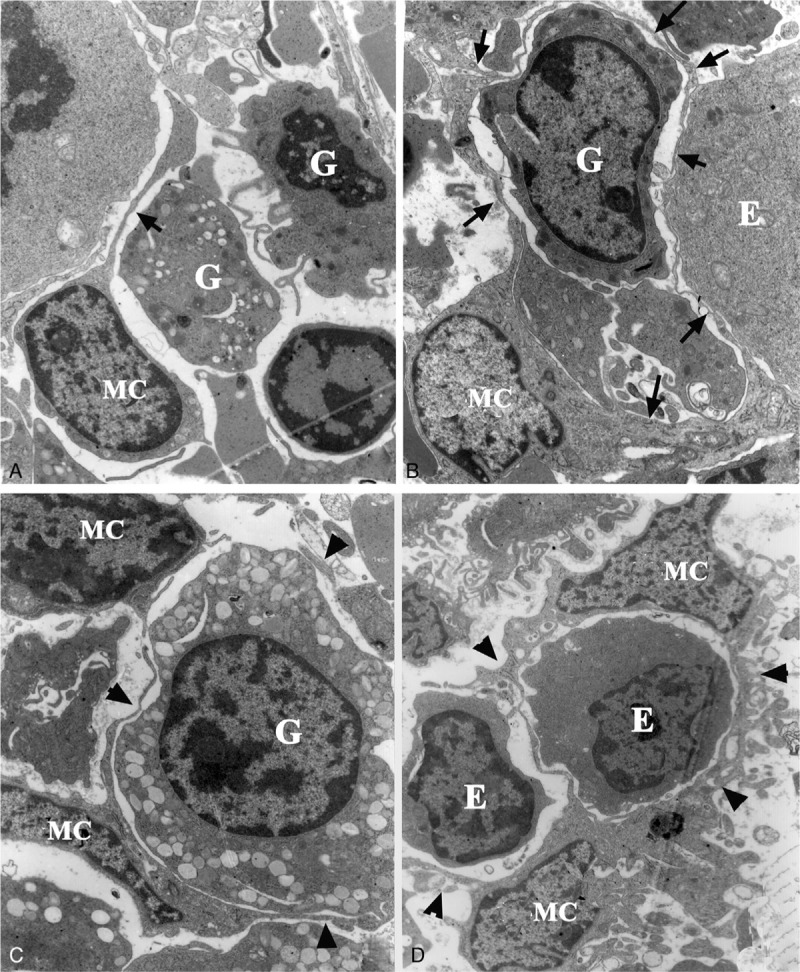
Ultrastructural characteristics of b-BMMCs (MC). (A) An undeveloped b-BMMC with fewer processes includes a prominent nucleolus and scarce cytoplasm (arrow) between a granulocyte (G) and an erythrocyte (E), ×2000; (B) a developed b-BMMC has many bifurcate processes (arrows) between hematopoietic cells, ×2500; (C) two b-BMMCs project fine processes (arrowheads) around a granulocyte (G), ×3000; (D) two b-BMMCs envelop and isolate erythrocytes by processes (arrowheads), ×2500.

**Figure 6:**
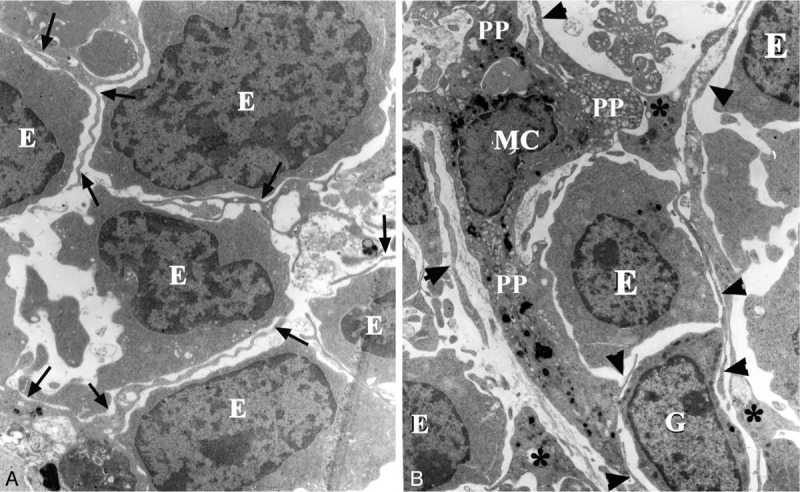
Bifurcate processes of b-BMMCs. (A) Bifurcated processes about 60 nm thick (arrows) isolate erythroblasts in various developmental stages (E), ×4000. (B) thick proximate processes (PP) of a b-BMMC (MC) contain more lysosomes in the cytoplasm, but thinner processes (arrowheads) are bifurcated at a locus (stars) among granulocytes (G) and erythrocytes (E), ×4000.

A few b-BMMCs shared undeveloped features with a prominent nucleolus and fewer processes around the main cell bodies (Fig. 5**A**), but most b-BMMCs were fully developed, characterized by a smaller main body with an ovoid nucleus and more bifurcated processes among hematopoietic cells. They included plentiful rER, lysosomes and phagosomes in the peripheral cytoplasm and proximate processes (Fig. 5**B**). Sometimes, a few b-BMMCs connected together and enclosed hematopoietic cells by processes (Fig. 5**C, D**). The processes usually divaricated hierarchically and became thinner and thinner between hematopoietic cells. Distal processes were from 60 to 80 nm thick and contained few cellular organelles, which constructed a honeycomb-like structure for hematopoietic cell in bone-marrow granules (Fig. 6).

### Ultrastructure of i-BMMCs on TEM

**Figure 7:**
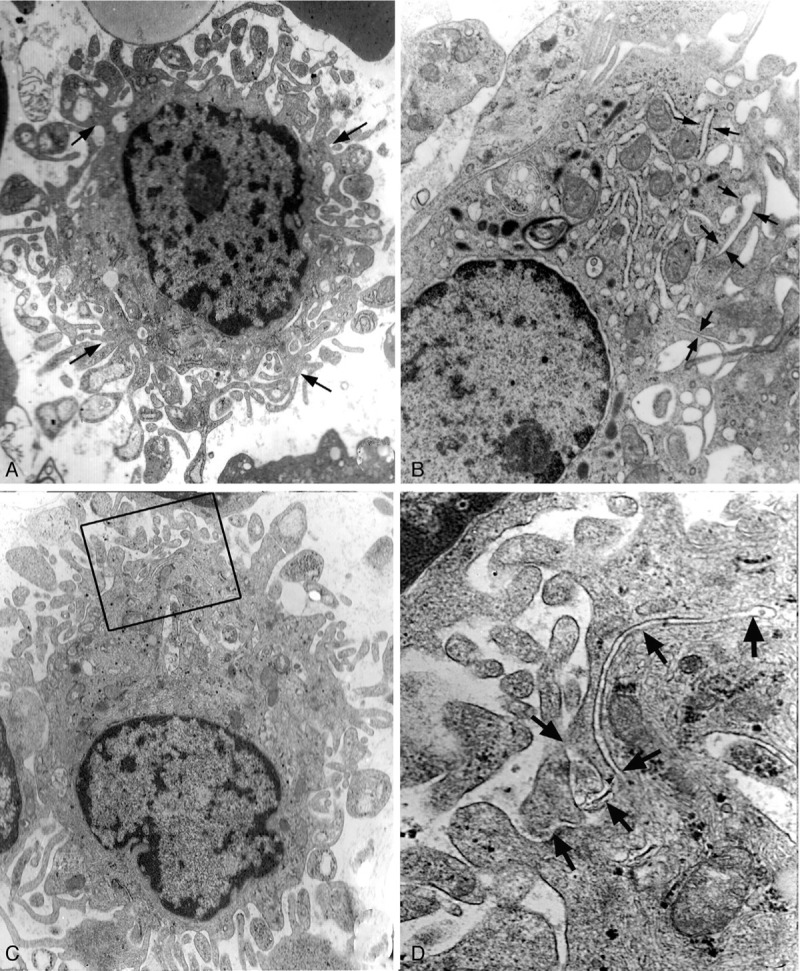
Ultrastructure of i-BMMCs. (A) An i-BMMC about 50 μm in diameter has numerous bifurcate parapodiums on its cell surface (arrows), ×2.5K. (B) an i-BMMC shows a meshwork feature resulting from rER expansion (pairs of arrows) in the peripheral cytoplasm, ×10K. (C) parapodiums connect with peripheral cytoplasm of an i-BMMC, ×3K. (D) a high magnification of (C), rER (arrows) demarcate cytoplasm into segments as large as parapodiums on the surface, ×30K.

**Figure 8:**
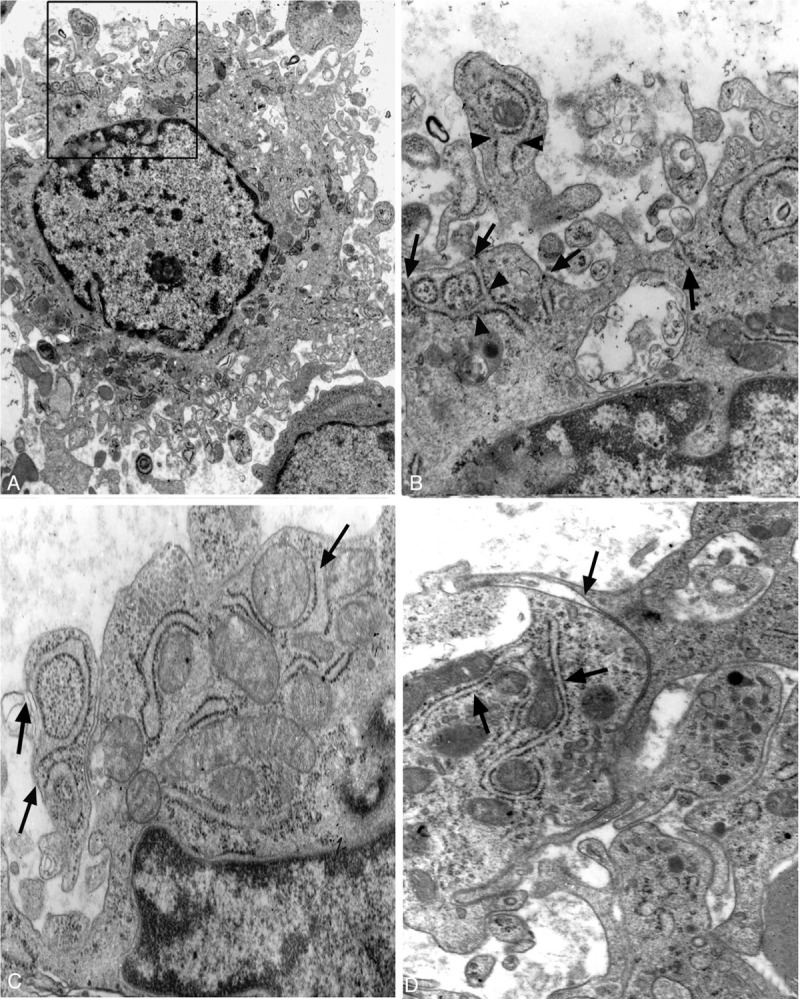
Ultrastructure of i-BMMCs. (A) An i-BMMC shows plentiful bifurcate parapodiums on the surface and contains more lysosomes in the cytoplasm, ×3K. (B) a high magnification of (A), rER segregates the peripheral cytoplasm (pair of arrows) and is connected with the surface parapodiums (arrows), ×30K. (C) many rER cisternae open on to the surface parapodiums (arrows), ×40K. (D) ER (arrows) demarcates cytoplasm into segments as large as the surface parapodiums, ×30K.

i-BMMCs were about 60 μm in diameter, including a round nucleus, plentiful lysosomes, rER, and many bifurcate parapodiums on the cell surface (Fig. 7**A, C** and Fig. 8**A**). Most rER cisternae were expanded and divaricated, demarcating cytoplasm into segments like those in megakaryocytes.^13,14^ The segments of cytoplasm were of the same size as parapodiums on the cell surface. In higher-power views, rER often appeared to open on to the cell surface and connect with the plasmalemma of i-BMMCs (Fig. 8**B–D**).

## DISCUSSION

Bone-marrow stromal cells (BMSCs) are a heterogeneous group of cells with distinctive morphological features that construct hematopoietic microenvironments by varied physical structures and releasing different hematopoietic factors in bone marrow. Each BMSC is located in a specific region, for example, osteoblasts, osteoclasts and chondrocytes line the endosteal surface, whereas adventitial cells and pericytes are adjacent to the outer wall of sinusoids and perivascular regions.^15–17^ As such, they compose dynamic hematopoietic microenvironments of HSPCs in bone marrow. BMMCs are pleomorphic depending not only on its inherent entity but also external factors.

In the present study, b-BMMCs showed irregular processes with blurred boundaries among hematopoietic cells; however, i-BMMCs exhibited a round nucleus and coarse processes with granular cytoplasm in bone-marrow smears. The above appearances were seldom described because of the difficulty in identifying them in obscure images in bone-marrow smears by light microscopy. In semithin sections of Epon 812 blocks, b-BMMCs exhibited the original morphology of those in vivo, characterized by a main body with an oval nucleus and many bifurcate processes projecting out between hematopoietic cells. In culture conditions, most c-BMMCs were spindle shaped, but some of them were flat. The flat BMMCs often adhered to hematopoietic cells and showed AKP activity in H4434 methylcellulose semisolid medium. All c-BMMCs in MEM media were flat and polygonal, and positive for CD44 and α-SMA. The results were consistent with other observations.^5^^,18^ The above appearances suggested that the morphology of BMMCs was affected predominantly by local microenvironments and culture conditions.

On TEM, b-BMMCs stretched out many bifurcate processes among hematopoietic cells, most of them about 60 nm thick, more than 3 times that of the plasmalemma, and constructed a honeycomb-like meshwork for hematopoietic cells in bone-marrow spaces. The question arises: how do b-BMMCs obtain the large amount of membrane to supplement the plasmalemma on these processes in bone marrow?

In attempting to answer this question, it should be noted that i-BMMCs had many parapodiums on the surface and plentiful rER in the cytoplasm compared with the processes and cytoplasm of b-BMMCs. The morphologic differences might be associated with external factors: parapodiums on i-BMMCs resulted from the release of hematopoietic cells and process retraction of b-BMMCs during bone-marrow aspiration. In contrast, processes of b-BMMCs resulted from the squeezing and pressing together of hematopoietic cells in bone marrow. Furthermore, detailed TEM images demonstrated that rER were bifurcated in the peripheral cytoplasm and connected with plasmalemma of i-BMMCs. It suggested that the huge plasmalemma of b-BMMCs may be derived from cytoplasmic rER. In other words, rER may contribute the membrane component and, in effect, be the origin of the processes of BMMCs in bone marrow.

Theoretically, plasmalemma is supplemented by the enveloping membrane of recycling phagosomes and vesicles from Golgi apparatus; membrane of the Golgi apparatus was mainly derived from rER during protein transport in well-developed cells.^19–22^ In fact, the conventional view has been that vesicles and phagosomes act predominantly as transporters or carriers of proteins rather than being a membrane resource for the plasmalemma.^23,24^

Cell morphology and structure was not only determined by cytoskeleton components of actin, tubulin, myosin, dynamin, actinin and supervillin filaments, but also was influenced by other cells in multicellular tissues.^25,26^ In particular, the morphology of b-BMMCs was influenced by environmental factors, that is, bifurcate processes were associated with a pressing together of hematopoietic cells. However, this specific network demands a large amount of membrane to supplement the plasmalemma of b-BMMCs in bone marrow. In this study, evidence has been provided for the phenomenon of prominent rER connecting with plasmalemma, which suggested that plasmalemmal expansion was closely related to transformation of rER in the cytoplasm of b-BMMCS in the same way as occurs in yeast cells.^27,28^

**Figure 9:**
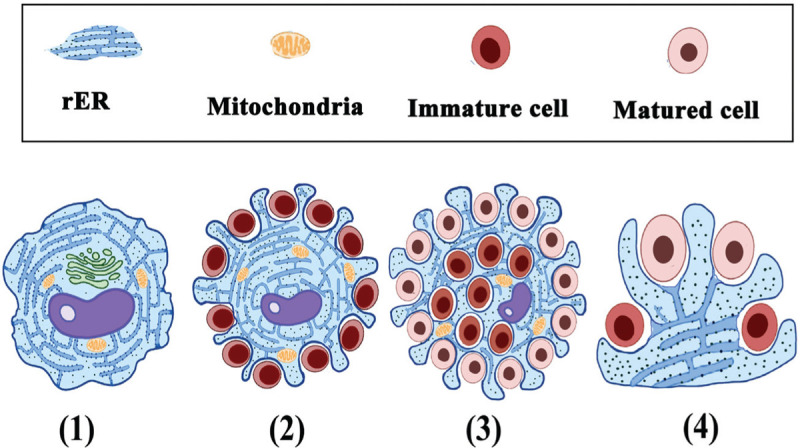
Diagrammatic representation of BMMC transformation associated with rER. (1) Isolated BMMCs without processes contain prominent rER, Golgi apparatus and mitochondria. (2) BMMCs send out short processes owing to the pressing together of undeveloped hematopoietic cells. (3) With hematopoietic cells maturation and proliferation, BMMCs extrude more bifurcate processes around varied hematopoietic cells in different developmental stages. (4) Model of rER transformation into plasmalemma on bifurcate processes of BMMCs.

As a multifaceted organelle, rER is transported between the Golgi apparatus and nuclear envelope through a stripping of membrane-bound ribosomes in mammalian cells.^29,30^ Some experiments have recently revealed that rER contacted the plasmalemma and participated in macrophage phagocytosis.^31–33^ Gagnon and colleagues^34^ showed that phagosome membranes were derived from rER in addition to plasmalemma during macrophage phagocytosis. The relationship between the plasmalemma and endoplasmic reticulum has also been investigated and identified in various cells in recent decades.^35–37^ These studies reinforce the presumption that rER of BMMCs may be transformed into plasmalemma in human bone marrow (Fig. 9).

## CONCLUSIONS

BMMCs show a degree of polymorphism dependent on their different environments. Their extensive slender processes appear to have a role in the development of adjacent hematopoietic cells, and the rER of BMMCs may in turn have a role as a membrane resource for the plasmalemma of these processes. Confirmation of the direct interaction between rER and plasmalemma awaits further high-resolution ultrastructural studies in the future.

## MATERIAL AND METHODS

### Light microscopy

All bone-marrow aspirates were from of 30 patients with hematocytopenia presenting at our hospital. Bone-marrow smears were prepared and surveyed according to our routine diagnostic schedule, but bone-marrow granules were prospectively collected from bone-marrow aspirates and processed for TEM. Semithin sections of bone-marrow granules in Epon 812 blocks were stained by the Wright-Giemsa method and observed by light microscopy.^11^

### BMMCs culture and cytochemical staining

Mononuclear cells were isolated from bone-marrow aspirates by lymphoprep following gradient centrifugation. Part of them was cultured in H4434 methylcellulose semisolid medium, and c-BMMCs were demonstrated on the bottom surface of dishes by Wright staining and the activity of alkaline phosphatase (AKP) was detected by cytochemistry after 14 days. Other mononuclear cells were incubated in MEM media with 10% fetal bovine serum for a week, and c-BMMCs on the bottom surfaces of dishes were also identified by monoclonal antibodies for CD44 and α-smooth muscle actin (α-SMA) immunocytochemically.^12^

### Electron microscopy

The c-BMMCs, adhering with hematopoietic cells on the bottom surfaces of dishes with MEM media, were processed for SEM. Mononuclear cells and bone-marrow granules isolated from bone-marrow aspirates were processed according to TEM procedures. Briefly, the samples were fixed in 2.5% glutaraldehyde, postfixed in 1% osmium tetroxide, washed in phosphate-buffered saline, dehydrated in graded alcohols and embedded in Epon 812. Ultrathin sections at 60 nm were stained with uranyl acetate and lead citrate. The b-BMMCs and i-BMMCs were observed by TEM.
